# Manufacturing and Management of CAR T-Cell Therapy in “COVID-19’s Time”: Central Versus Point of Care Proposals

**DOI:** 10.3389/fimmu.2020.573179

**Published:** 2020-10-15

**Authors:** Iñaki Ortiz de Landazuri, Natalia Egri, Guillermo Muñoz-Sánchez, Valentín Ortiz-Maldonado, Victor Bolaño, Carla Guijarro, Mariona Pascal, Manel Juan

**Affiliations:** ^1^Department of Immunology, Centre de Diagnòstic Biomèdic, Hospital Clínic de Barcelona, Barcelona, Spain; ^2^Department of Hematology, Institut Clínic de Malalties Hematològiques i Oncològiques, Hospital Clínic de Barcelona, Barcelona, Spain; ^3^Institut D’Investigacions Biomèdiques August Pi i Sunyer, University of Barcelona, Banc de Sang i Teixits – Hospital Clínic de Barcelona Immunotherapy Platform, Barcelona, Spain; ^4^Allergy Network ARADyAL, Instituto de Salud Carlos III, Madrid, Spain; ^5^Hospital Sant Joan de Déu, Esplugues de Llobregat, Barcelona, Spain

**Keywords:** chimeric antigen receptor, adoptive cell immunotherapy, SARS-CoV-2 coronavirus, manufacturing process, good manufacturing practice

## Abstract

The COVID-19 pandemic, caused by Severe Acute Respiratory Syndrome Coronavirus-2 (SARS-CoV-2), has generated a significant repercussion on the administration of adoptive cell therapies, including chimeric antigen receptor (CAR) T-cells. The closing of borders, the reduction of people transit and the confinement of the population has affected the supply chains of these life-saving medical products. The aim of this mini-review is to focus on how the COVID-19 pandemic has affected CAR T-cell therapy and taking into consideration the differences between the large-scale centralized productions for the pharmaceutical industry versus product manufacturing in the academic/hospital environment. We also review different aspects of CAR T-cell therapy and our managerial experience of patient selection, resource prioritization and some practical aspects to consider for safe administration. Although hospitals have been forced to change their usual workflows to cope with the saturation of health services by hospitalized patients, we recommend centers to continue offering this potentially curative treatment for patients with relapsed/refractory hematologic malignancies. Consequently, we propose appropriate selection criteria, early intervention to attenuate neurotoxicity or cytokine release syndrome with tocilizumab and prophylactic/preventive strategies to prevent infection. These considerations may apply to other emerging adoptive cell treatments and the corresponding manufacturing processes.

## Introduction

The SARS-CoV-2 coronavirus has generated an unprecedented global impact on multiple aspects of society, economy and health. SARS-CoV-2 was reported as a new emerging zoonotic pathogen in Wuhan (China) in December 2019, and declared as a pandemic by the World Health Organization in March 2020 (1). Its rapid human-to-human transmission has affected millions of people (2). Indeed, the routine operation of medical systems has been significantly disrupted generating a health crisis in many countries due to limited resources such as hospital beds and personal protective equipment. In early June, the number of infected people had already risen to more than 6 million globally and the number of new cases detected is increasing daily. There is not pre-existing specific immunity placing at risk humanity as a whole to infection by SARS-CoV-2. Taken together with the severe pulmonary and systemic inflammatory complications associated with the disease, it has caused governments to enforce isolation measures to prevent its rapid spread. Hospitals have been forced to change their usual workflows to cope with the saturation of hospitalized services. The closing of borders, the reduction of the transit of people and the confinement of the population has affected the supply chains of products. Access to life-saving drugs has also been affected, negatively impacting on patients with other life-threatening diseases (3).

Chimeric antigen receptor (CAR) T-cell therapy is a life-saving bioengineered cell replacement therapy against leukemia. This adoptive antitumor immunotherapy, based on autologous T-cells transduced with a genetically engineered receptor for CD19 to redirect their cytotoxicity against native CD19 surface antigens expressed in tumor cells, has completely changed the management of patients with hematologic malignancies such as acute lymphoblastic leukemia (ALL) or non-Hodking lymphoma (NHL) (4–6). Tisagenlecleucel (Kymriah, Novartis) and axicabtagen ciloleucel (Yescarta, Kite/Gilead) are both anti-CD19 CAR T-cell (CART19) commercial products that obtained Food and Drug Administration (FDA) approval in 2017 for the treatment of pediatric and young adult patients with CD19+ relapsed/refractory B-cell ALL, relapsed/refractory B-cell NHL [diffuse large B-cell lymphoma (DLBCL), primary mediastinal B-cell lymphoma and transformed follicular lymphoma]. Other regulatory agencies like the European Medicines Agency (EMA) have also recently approved these commercial CART19 use. Therefore, CART19 is considered a potential curative therapeutic option for CD19+ hematologic malignancies with no response to conventional treatments. In addition, approval of other CAR T-cell products developed commercially against other molecular targets are expected as further options for treatment in the coming months. More than 500 clinical trials^1^ are being carried out testing CAR-based products. Some of them are financed by the industry and others are being developed in the academic ambit. The cohabitation of both is inevitable and necessary. Industry production could supply CAR T-cell therapies to reach the whole population using good manufacturing practice (GMP) accredited large facilities to centralize the manufacturing process. On the other hand, academic centers should be dedicated to develop CAR T-cell therapies against less frequent diseases. Independently, both have been seriously affected by the COVID-19 pandemic despite the differences they present in product manufacturing and management processes (7). The management of CAR T-cell therapy is a complex time-consuming process that requires highly specialized personnel and coordinated work systems including medical management of the patient before and after the infusion due to feasible toxicities (8–10).

Owing to the doubtless relevance of CAR T-cell therapy for hematologic patients, it is important to have in mind some considerations within the context of the COVID-19 pandemic. Therefore, the aim of this mini-review is to focus on how the effects caused by the pandemic have affected this therapy taking into consideration the differences between the large-scale centralized production of CAR T-cells by the pharmaceutical industry versus the product manufacturing processes employed by the academic/hospital environment. We also review different aspects of CAR T-cell therapy, including patient selection and resource prioritization performed in our center during the COVID-19 pandemic.

## Cautious Continuation vs Deferment of CAR T-Cell Therapy During COVID-19 Pandemic

Chimeric antigen receptor T-cell therapy has elicited an unprecedented response against B-cell malignancies, but it is associated with significant toxicity, including prolonged cytopenia, cytokine release syndrome (CRS), and neurotoxicity (10–13). Toxicity is normally associated with the T-cell’s inherent mechanism of action that has been well-described in academic and industry developed CAR T-cells (14). The use of CART19 cell therapy can result in prolonged B-cell aplasia and therefore inability to develop an antibody response necessary to respond against pathogens such as SARS-CoV-2 (15, 16). Furthermore, these patients are usually managed as in-patients to facilitate intensive monitoring due to the severity and rapidity of CRS, neurotoxicity and frequent need the intensive care unit (ICU) (17).

The COVID-19 pandemic is a threat to interrupt any cell therapy. It is priority that each center carefully review the internal policies and procedures to adopt the recommendations needed according to their healthcare needs. Hospitals have instituted measures to defer multiple medical interventions, including adoptive cell treatments or hematopoietic stem cell transplantation. Another relevant consideration is the inconsistent uniformity of clinical cell therapy protocols. This hampers the establishment of standardized strategies during the COVID-19 pandemic. A large proportion of these patients receive therapy in academic or pharmaceutical clinical trials and many stopped to preserve patient’s safety. Indeed, lymphodepletion before final cell-product infusion facilitates the expansion of CAR T-cells as it generates a suitable environment for *in vivo* modified T-cell expansion and survival (18). However, this generates a severe immunosuppression that can be seriously complicated by SARS-CoV-2 infection. Nevertheless, delaying cell therapy as a consequence of the COVID-19 pandemic could be fatal for the majority of patients with relapsed/refractory malignancies. Alternative therapeutic strategies are also generally associated with significant immunosuppression and subsequent potential hospitalizations. In fact, CAR T-cells are potentially curative for patients with poor prognosis (19).

### Required Resources for Safe Cell Therapy During the COVID-19 Pandemic

Not all centers will be affected in the same way during the COVID-19 pandemic. Therefore, a careful evaluation of resources and infrastructures should be performed between the department of hematology and the hospital emergency planning group. The availability of ICU, hospital beds and mechanical ventilation equipment are limited, but required resources. Moreover, staff shortages due to potential exposure and resource constraints on personal protective equipment affect the management of the patient before and after the treatment infusion.

Some practical aspects should be considered for the safe administration of CAR T-cell therapy during the pandemic (Table 1):

• In the case of ICU collapse: establish a triage algorithm to select only patients who are most likely to benefit with no alternative treatment options and in whom the risk of toxicity is lower.
• Guarantee the availability of personnel to perform the leukapheresis and the reception and sample processing in the adoptive cellular immunotherapy unit.
• Initiate lymphodepletion procedure only after receiving CAR T-cell products at the site to avoid potential obstacles on supply chain operations.
• Hospital bed availability should be ensured for the immediate 4 weeks surrounding the treatment. There are few children admitted with COVID-19 so young adult patients who may benefit from this therapy may be transferred to a Pediatric Center with greater availability of beds.
• Guarantee the availability of a member of the medical team with the capacity to respond to complications associated with COVID-19.
• Establish a specific workflow for patients infected with COVID-19.

**TABLE 1:** Practical aspects before and after the manufacturing process for the safe administration of an academic CAR T-cell therapy during the COVID-19 pandemic.

**Before the manufacturing process of CAR T-cells**	
Patient selection	Selection of appropriate patients
	Clear assessment of risk/benefit balance
Screening measures	Assess for signs/symptoms of COVID-19
	Evaluation of risky contacts at relevant time points, including before leukapheresis and before CAR T-cell infusion
	Laboratory qPCR testing for SARS-CoV-2 for every patient before leukapheresis
ICU capacity	Guarantee the availability of ICU beds
	Young adult patients may be transferred to a Pediatric Center to ensure availability of ICU beds
Working protocols	Establish a specific workflow for the management of patients infected with SARS-CoV-2
Availability of personnel	Guarantee the availability of a member of the medical team with the capacity to respond to complications related to COVID-19
**After the manufacturing process of CAR T-cells**	
Specific measures for infected patients	Final cell product should be tested for SARS-CoV-2 by qPCR before infusion in patients with a positive qPCR prior to leukapheresis
	The manufacturing process would continue if a patient becomes infected after leukapheresis. The infusion should be postponed until patient’s clinical improvement

### Patient Selection in the COVID-19 Pandemic Setting

The COVID-19 pandemic has altered inclusion algorithms for the proper selection of patients, that is now associated to ethical dilemma. It is imperative to outline criteria to identify optimal candidates who have the potential to achieve significant remission. The benefit-risk balance should be clear, taking into account the risk of delaying CAR T-cell therapy against the risk of progression of the underlying disease. Therefore, a group of experts should evaluate the inclusion of patients considering, among multiple factors, the lack of avalability in the hospital and alternative treatments if complications appear. In our experience at Hospital Clínic de Barcelona, a medium-sized academic institution, we have continued to administer our academic and other commercial CAR-T cell therapy against aggressive relapsed/refractory B-cell NHL and ALL (20, 21).

## Cellular Therapy Support During the COVID-19 Pandemic

During the first month of the COVID-19 state of alarm declared in Spain, every CAR T-cell operation was postponed and only one infusion was performed in our center. Ultimately, no treatment was canceled, although the final product infusions were delayed due to the lack of availability of space in the ICU. Different supportive measures to mitigate the risk of COVID-19 in our patients have been taken (Table 1). Two young patients (aged 26 and 30) were treated in another major hospital of the city, Sant Joan de Déu Children’s Hospital Center. After the first month of the pandemic, one bed at ICU was reserved for CAR T-cell therapy. This allowed the treatment of nine patients at Hospital Clínic de Barcelona. They were patients under the age of 70 with few co-morbidities and 0–2 ECOG functional status. Altogether, six patients were treated with academic CART19 and one patient with CAR T-cell therapy targeting BCMA (20). On the other hand, two patients were treated with CAR-T cells developed by the industry. The feasible impact of the delay on CAR T-cell treatment outcomes will be analyzed in the medium-long term.

### CAR T-Cell Guidance: Screening and Preventive Measures

SARS-CoV-2 viral detection tests have been performed in every patient treated with CAR T-cells at various time-points. Before leukapheresis, patients were screened for SARS-CoV-2 by qPCR given that qPCR is the gold standard test to measure viral loads. The determining factor in the start of the cell therapy manufacturing process is the infection status of the patient and the potential ability to transmit the virus. Serological tests report directly on humoral immunization but serology is not a specific marker of infection. A positive IgM result in the serological test is not always equivalent to an acute infection since it could persist for months without viral load being detected in the patient. As with other infections, serological tests are very useful for screening but should only be used for diagnosis if a gold standard test is not available.

Our patients were assessed for specific symptoms during the whole process. In addition, in-person visits have been avoided as a preventive measure and patients were asked about their potential risky contacts. The major importance of extreme confinement measures has been therefore repeatedly emphasized. The use of EPI facemasks in patient’s environment was essential to maximally reduce infection risk. The final cell product should be tested for SARS-CoV-2 by qPCR before infusion in those patients with a positive qPCR prior to leukapheresis.

The special committee formed in our center to evaluate SARS-CoV-2-positive cases initially decided to avoid the CAR T-cell manufacturing process if COVID-19 was diagnosed in order to reduce possible contagions among medical staff and contamination of manufacturing facilities. This decision was controversial considering the low risk of contamination in a closed-circuit manufacturing process and the low viral load in blood samples (22). Currently, we have established to perform the leukapheresis 12 days after a positive qPCR result to ensure that the viral load in patient’s samples are virtually undetectable (23).

Diverse complications that could emerge from SARS-CoV-2 infection could actually worsen CAR T-cell therapy outcomes. Thorough monitoring of these patients is required in order to detect eventual neutropenia or other infection. Post-infusion standard antiviral, antifungal and antimicrobial prophylaxis protocols were recommended.

## Manufacturing Time for Academic vs Pharmaceutical CAR T-Cell Therapies

**FIGURE 1:**
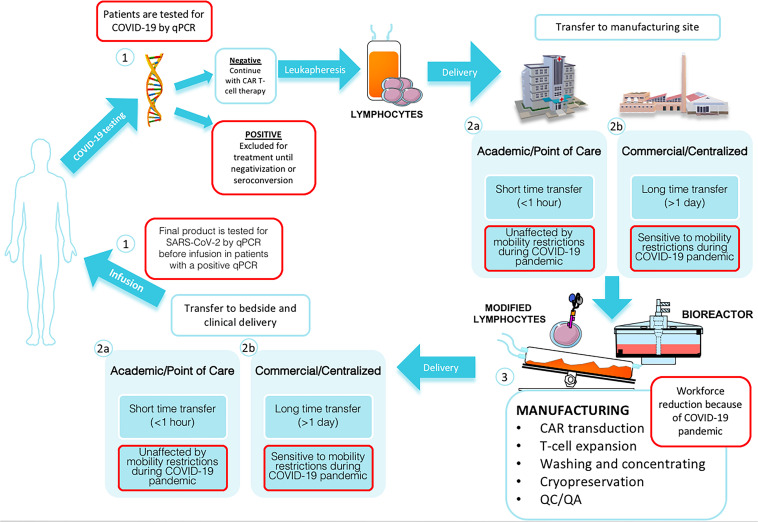
Standardized CAR T-cell manufacturing process during COVID-19 pandemic. Patients have to be tested by qPCR for SARS-Cov2 before leukapheresis (1); in case of a negative result, peripheral blood mononuclear cells (PBMCs) are collected from patient via leukapheresis. Timing of delivery to manufacturing site and the susceptibility to mobility restriction varies between Academic/Point of Care (2a) and Commercial/centralized (2b) production. Manufacturing process is affected by workforce reduction (3). T-cells are selected, activated, transduced with a viral or non-viral vector to express the desired CAR and expanded in a bioreactor. After that, resulting CAR T-cells are isolated and cryopreservated and assessed by quality control (QC) and quality assurance (QA) programs. Finally, the patient’s modified own cells are transferred to bedside. Timing of delivery to bedside and the susceptibility to mobility restriction varies between Academic/Point of Care (2a) and Commercial/centralized (2b) production. Before CAR T-cell product infusion, final product is tested for SARS-CoV-2 by qPCR before infusion in patients with a positive qPCR result prior to leukapaheresis (1).

Autologous CAR T-cell therapy requires a personalized manufacturing process based on several critical steps that demands good coordination between different medical disciplines. For those heavily treated patients, time is crucial and well-established workflows are essential for academic or commercial cell-based therapies. After the theoretical design of the synthetic chimeric receptor and the corresponding pre-clinical studies, the complete process for CAR T-cell therapy includes (i) obtaining the starting patient’s cell population by leukapheresis followed by the (ii) *ex vivo* genetic introduction of the synthetic CAR into these cells using mainly lentiviruses or retroviruses, previously generated. These modified T-cells are then (iii) activated and expanded in bioreactors and the resulting product might be (iv) adequately prepared and cryopreserve in infusible media for the final (v) product re-infusion into the patient (24, 25). All these steps can be affected by the pandemic setting. Along with inherent complexity in any cell therapy, limitations and obstacles in academic hospitals and pharmaceutical industry must be added. In this way, the standard manufacturing period of 9–11 days until the final product could be fatally extended (Figure 1).

### COVID-19 Impact to Delivery and Critical Supply Chains

The main difference between academic and pharmaceutical CAR T-cells, regarding the timeline of the manufacturing process, is obtaining the “living drug.” As for academic products, commercial CART19 are based on autologous T-cells and each patient requires his own T-cells. In the academic CAR T-cell manufacturing, all steps are carried out in the hospital facilities enabling a more flexible, personalized and coordinated “patient-friendly process.” It allows short transfer times from the leukapheresis site to the production facilities and from the production center to the patient’s bed, both being less than 1 hour (26). In commercial manufacturing, the same process takes place in distant geographical locations and depends on delivery protocols established by pharmaceutical companies. Logically, the transfer times from the leukapheresis site to large-scale production GMP facilities and from this production center to the patient’s bed could be, at least, 1 day (27).

However, COVID-19 has rapidly constrained travel and mobility, extending the delivery times of commercial CAR T-cell products. Another side effect of reduced mobility are the potential disruptions in the resources supply line essential to the manufacturing process. This may affect both academic CAR T-cell products and those produced by industry. For example, the parallel process to obtain vectors with the CAR transgene like lentiviruses is a compendium of GMP skilled steps where different reagents are needed. Similarly, other specialized products are essential for the activation and expansion of T-cells in a bioreactor like CliniMACS Prodigy (28, 29). These critical steps can be seriously aggravated in countries where the production of these reagents is scarce and are imported from other regions. However, the large-scale production that takes place by industrial facilities ensures a greater storage capacity and a robust supply chain.

### COVID-19 Impact to Availability of Personnel in Every Step of the Manufacturing Process

The reduction of personnel due to possible contagions among the staff, the restructuring of the workforce to treat infected patients and the limited resources and protective equipment has been a cause for concern (Table 1). All of these factors can impact equally on the academic or pharmaceutical production modalities.

The staff and the reagent shortage can affect the leukapheresis process and cell processing in the laboratory. There exist strict regulations associateds with cell therapy where personnel who develop “living drugs” must work under sterile and GMP conditions. For this reason, a contagion that could spread between qualified personnel and the consequent imposing quarantine that would be imposed on the rest of the personnel can become a bottleneck in the global production of the adoptive cell therapy. The stringent and immobile regulation has not been amended in the context of the COVID-19 pandemic to ensure the safety of patients, medical staff and laboratory personnel.

Similarly, within the associated legislation, a series of quality controls are imposed and must be complied with before CAR T-cell product infusion. Among them, the confirmation of the product sterility is essential. In addition, it must be ensured that the product is free of endotoxins, adventitious viruses and mycoplasma. There are other non-microbiological controls that must be carried out as the number of copies of the CAR per genome, flow cytometry experiments to determine the percentage of cells with CAR, among others. Having these controls in place for each cell product requires precious time in COVID-19 pandemic setting and is highly dependent on human work. Therefore, any delay in verifying all of these control points may delay the infusion.

## Cytokine Release Syndrome and Tocilizumab Prioritization

Once the manufactured CAR T-cell product is infused, a resulting CRS is the widest described associated side effect. A compilation of different symptoms from hypotension to fever due to a massive pro-inflammatory cytokine release (IL-6, IL-1β, TNF-α) have been reported (30). Serum IL-6 level has been correlated with CRS severity (15). Hence, tocilizumab (TCZ), an anti-IL-6 receptor monoclonal antibody, has become the drug of choice for the management of moderate or severe CRS (10). This IL-6 cytokine release profile resembles the virally driven hyper-inflammation (CRS-like) suggested as predictor of fatality in COVID-19 (31). This fact has led to the setting-up of several clinical trials with the aim of studying the impact of TCZ administration in the evolution of the acute respiratory distress syndrome and the off-label use of this drug in symptomatic COVID-19 patients.

The management of CRS is extremely relevant given that viral infections (not only COVID-19) may promote CRS (32). CRS requires treatment with TCZ or corticosteroids depending on severity of symptoms. The notable increase in TCZ use has raised a concern about supply during COVID-19 pandemic. However, availability of 2 TCZ doses for each patient is a mandatory condition before CAR-T infusion. Two patients treated with CAR T-cell therapy have received TCZ to treat CRS in our center in the pandemic setting. Tocilizumab administration protocol has not been reconsidered despite supply problems. This protocol consists on early TCZ use at the onset of grade 2 CRS. We have dealt with TCZ scarcity through the use of other drugs such as sarilumab (human anti-IL-6 receptor) or siltuximab (chimeric anti-IL-6) to face the CRS-like complications. If ICU is collapsed, CRS management protocols should initiate earlier to reduce the probability of using ICU. As a practical option, we propose TCZ administration from grade 1 CRS (and not grade 2 as is usually done), or even prophylactic TCZ administration in those patients considered to be at high risk of severe CRS, with high tumor burden. Corticosteroids used as CRS treatment when TCZ has failed could promote potentially disadvantageous effects on COVID-19 outcomes (33). Nevertheless, corticosteroids should be cautiously used in COVID-19 patients that suffer from CRS after CAR T-cell infusion (34).

In fact, the current pandemic has exposed us to an ethical dilemma regarding the prioritization of TCZ. Each case must be thoroughly evaluated to ensure the availability of this drug. Tocilizumab could decrease the duration and severity of COVID-19 symptoms allowing the weaning of the ventilatory support (34). On the other hand, TCZ supply prior to infusion must be ensured for the hematologic patients during CAR T-cell therapy to mitigate the toxicity of an eventual CRS complication.

## Conclusion

CAR T-cell therapy has been consolidated as a potential curative therapy for patients with refractory/relapsed hematologic malignancies. The COVID-19 pandemic represents an unprecedented challenge to continue safely treating patients with this adoptive cell therapy. Nevertheless, centers should continue offering this potentially curative treatment with CAR T-cell therapy for critical patients using appropriate selection criteria, early intervention to attenuate side effects like CRS with standardized TCZ protocols, and prophylactic/preventive strategies to prevent infection. For those heavily treated patients, time is crucial. Mobility and personnel restrictions add obstacles to a truly personalized therapy in which multidisciplinary teams intervenes. This has affected both academic CAR T-cell therapies and those commercially available. There are several limitations related to delivery and the time-consuming manufacturing processes that create new concepts concerning the benefit of academic point of care proposals. Also, some regulatory rules should be re-evaluated to become more flexible during this pandemic.

## Author Contributions

MJ and MP proposed, directed, discussed, and revised the development of the manuscript. GM-S, NE, and IOL had equally contributed to the data gathering and writing of the review. VO-M reviewed and collaborated particularly on aspects related to the hematological issue. VB and CG had been in charge of analyzing the technical aspects related to the product manufacturing. All authors contributed to the article and approved the submitted version.

## Conflict of Interest

VO-M is a recipient of a research grant from Fundación Española de Hematología y Hemoterapia (FEHH), also declares travel grants (Kite, Celgene, Novartis, Roche, Takeda and Janssen), consultant or advisory fees (Kite, Celgene, and Novartis) and honoraria (Kite). The remaining authors declare that the research was conducted in the absence of any commercial or financial relationships that could be construed as a potential conflict of interest.

## Footnotes

1 www.clinicaltrials.gov

## Abbreviations

SARS-CoV-2: Severe Acute Respiratory Syndrome Coronavirus-2
CAR: chimeric antigen receptor
ALL: acute lymphoblastic leukemia
NHL: non-hodking lymphoma
CART19: anti-CD19 CAR T-cell
FDA: Food and Drug Administration
DLCL: diffuse large B-cell lymphoma
GMP: good manufacturing practice
CRS: cytokine release syndrome
ICU: Intensive Care Unit
TCZ: tocilizumab
PBMCs: peripheral blood mononuclear cells.
